# Generative Artificial Intelligence Use and Adolescent Mental Health

**DOI:** 10.1007/s40124-026-00388-8

**Published:** 2026-09-21

**Authors:** Jason M. Nagata, Jacqueline O. Hur, Thang Diep, Hasan Alsamman, Eliot D. Lee, Alexander Heuer, Henry Huynh, Sahana Nayak, Ryan McBain, Jonathan Cantor, Jason M. Lavender

**Affiliations:** 1 Department of Pediatrics, University of California, San Francisco, 550 16th Street, 4th Floor, Box 0503, San Francisco, CA 94143 USA; 2 RAND, Arlington, VA USA; 3 RAND, Santa Monica, CA USA; 4 Military Cardiovascular Outcomes Research Program (MiCOR), Department of Medicine, Uniformed Services University of the Health Sciences, Bethesda, MD USA; 5 The Metis Foundation, San Antonio, TX USA

**Keywords:** Generative Artificial Intelligence, AI, Adolescent, Mental health, Problematic AI use, Psychopathology

## Abstract

**Purpose of Review:**

To synthesize recent literature on the relationships between generative artificial intelligence (GenAI) use and adolescents’ mental health, with particular attention to problematic use and psychiatric vulnerabilities.

**Recent Findings:**

Emerging evidence suggests associations between GenAI use and a range of adolescent mental health outcomes, including depression, anxiety, psychosis-related experiences, externalizing behaviors, eating disorder risk, and suicidality. Problematic patterns of use, including dependency and emotional reliance, may be particularly relevant among adolescents with preexisting psychiatric vulnerabilities. Current evidence suggests that these relationships may be bidirectional: psychological distress can motivate GenAI use for companionship, emotional support, or escape, while problematic use may reinforce maladaptive beliefs, displace real-world relationships, and amplify existing vulnerabilities. However, evidence remains predominantly cross-sectional, limiting causal inference.

**Summary:**

GenAI may interact with and amplify underlying psychiatric vulnerabilities during adolescence, highlighting the importance of understanding patterns and motivations of use. Pediatricians and other clinicians caring for adolescents could consider not only the frequency of GenAI use but also motivations for use, problematic dependency, and socioemotional reliance. Identifying high-risk usage patterns of GenAI through longitudinal research, particularly among youth with preexisting psychiatric risks, may inform the development of tailored screening, intervention, and prevention strategies.

## Introduction

Generative artificial intelligence (GenAI) is rapidly becoming integrated into the daily lives of many adolescents. Data reveal that approximately one-third of youth aged 4–17 years report daily use, and a subset engage with these platforms for extended periods each day [1]. Unlike traditional chatbots or recommendation algorithms that primarily rely on retrieval of existing information, GenAI creates novel content, including humanlike text, photorealistic images, and conversations [2, 3]. Adolescents can access GenAI not only through popular platforms such as ChatGPT and Gemini but also through embedded AI features within digital applications such as Snapchat’s “MyAI.” As these platforms become increasingly accessible, concerns have emerged regarding their influence on adolescent development, perceptions, and behavior [3].

This rapid uptake of GenAI is occurring against the backdrop of a worsening youth mental health crisis. Approximately 62.5% of mental disorders emerge before the age of 25, with a peak age of onset of 14.5 years, highlighting adolescence as a particularly vulnerable developmental period marked by social, cognitive, and neurobiological changes [4]. These early-onset conditions can have enduring consequences for psychological well-being, educational attainment, and social functioning [5–9], emphasizing the urgent need to identify modifiable factors, including patterns of GenAI engagement, that may influence mental health during adolescence and emerging adulthood.

Although the mental health implications of adolescent social media and screen use continue to be thoroughly investigated, GenAI introduces several novel features that may uniquely impact psychosocial functioning [10, 11]. Unlike traditional digital media, GenAI systems can provide highly personalized and engaging interactions and may exhibit sycophantic behavior as a byproduct of training optimization [12], increasing the risk of excessive or problematic usage. Recent high-profile media reports and a shifting policy environment have highlighted concerns about possible links between this rapid adoption of GenAI use and adverse mental health outcomes, including reports of suicidality, psychosis, social withdrawal, and maladaptive emotional reliance [13]. Adolescents may be particularly susceptible to these risks, as they may not always recognize when they are interacting with AI systems and may be more likely to accept AI-generated information without critically evaluating its accuracy, limitations, or underlying objectives [2].

Given the relatively recent launch of consumer-facing GenAI, the nascent literature on the correlates and potential effects of consumer-facing GenAI use is somewhat limited. In educational settings, in particular, ongoing research uses self-reported data to characterize how and why adolescents use GenAI [14], while other works investigate the utility of deploying structured AI tools to support learning [15] and how such tools compare with consumer-facing GenAI [16]. Existing research examining associations between GenAI use and mental health has focused largely on adult populations [17]. Within adolescent populations, research on GenAI has primarily centered on the use of machine learning and AI-based tools for mental health screening, prediction, and treatment rather than on adolescents’ direct interactions with consumer-facing GenAI systems [18]. Importantly, consumer GenAI platforms differ substantially from AI-based therapeutic interventions [19], which are typically highly controlled and designed with clinical oversight [20, 21]. Despite these differences, it has been estimated that nearly one in five adolescents report using consumer-facing GenAI tools for emotional support or mental health guidance [22]. Emerging findings regarding publicly available GenAI platforms have raised concerns about inadequate crisis response, risk assessment, and limited transparency regarding user privacy protections and model training practices [23]. A developmental perspective is particularly important because the relational and personalized features of GenAI intersect with ongoing changes in identity, social relationships, emotional regulation, and autonomy during adolescence.

This narrative review aims to describe the concept of problematic patterns of consumer-facing GenAI use, paralleling addiction-/dependence-based conceptualizations of digital media and social media use [24], as well as synthesize emerging evidence regarding the relationship between GenAI use and mental health among adolescents. We also propose a conceptual framework describing potential mechanisms through which GenAI may uniquely influence adolescent mental health outcomes. Finally, we identify key gaps in the existing literature and discuss practical considerations for pediatricians, mental health professionals, and other clinicians caring for adolescents, including approaches for assessing and addressing GenAI use in adolescent populations.

## Problematic and Dependent GenAI Use

Various forms of internet and digital media use have been examined within behavioral addiction frameworks. Gaming disorder is recognized in the International Classification of Diseases, 11th Revision (ICD-11), while Internet Gaming Disorder is included in the Diagnostic and Statistical Manual of Mental Disorders, Fifth Edition, Text Revision (DSM-5-TR) as a condition for further study [24]. Increasing GenAI use among adolescents and young adults has raised concerns over the development of dependence-like maladaptive engagement patterns characterized by loss of control, functional impairment, psychological distress, and continued use despite negative consequences [24]. Qualitative studies have already described adolescents and young adults experiencing behaviors and emotions consistent with problematic GenAI use, including emotional attachment, withdrawal-like distress, tolerance, relapse, sleep disruptions, social withdrawal, and difficulty reducing use [25, 26].

Problematic GenAI use may differ from traditional digital media addictions in important ways. Personalization enhances perceived relevance, immediate responses fuel instant gratification, and continuous engagement enables sustained interaction, all of which uniquely reinforce usage and dependence [24, 27]. GenAI systems are optimized through Reinforcement Learning from Human Feedback (RLHF), which allows AI systems to learn from human preferences by having users choose responses perceived as more helpful and satisfying [28]. However, this design renders GenAI structurally vulnerable to sycophantic behavior, characterized by excessive flattery and unwarranted affirmations [28]. Because RLHF can favor responses perceived as appealing or validating, sycophantic outputs may reinforce users’ existing beliefs and perceptions [28]. Studies have found that even a single interaction with sycophantic AI increased participants’ self-conviction and reduced participants’ willingness to accept responsibility during interpersonal conflicts [29]. The anthropomorphic design of conversational GenAI further encourages users to perceive these systems as empathic and socially meaningful [30]. Greater perceived anthropomorphism and empathy have consistently been associated with stronger emotional attachment and more problematic use of GenAI [19, 24].

Several theoretical frameworks may explain why adolescents may be particularly susceptible to problematic GenAI use, paralleling conceptualizations applied to problematic internet use, social media engagement, and other digital media technologies [31]. For instance, uses and gratifications theory proposes that individuals engage with media to satisfy psychological and social needs such as companionship, emotional support, entertainment, information seeking, and escape [32], whereas the compensatory use model suggests that digital technology may be used to cope with psychosocial distress or unmet interpersonal needs [1]. These motivations may be particularly salient during adolescence, a developmental period characterized by heightened sensitivity to peer relationships, identity formation, and emotional reactivity [33]. Ongoing maturation of prefrontal regulatory networks (i.e., self-regulation) alongside heightened limbic responsiveness (e.g., emotional intensity/reactivity) may further increase susceptibility to reinforcing AI interactions [34], particularly among youth with preexisting mental health vulnerabilities [35].

Behavioral addiction models similarly suggest that continuous emotional validation and personalized feedback may reinforce compulsive engagement, contributing to social withdrawal, sleep disruption, and increased psychological dependence that may exacerbate adverse mental health symptoms [24, 36]. Moreover, cognitive offloading theory suggests that adolescents may increasingly delegate cognitive tasks, decision-making, and academic work to AI, reinforcing both emotional and cognitive reliance [37]. Together, these mechanisms suggest that problematic GenAI use extends beyond excessive screen time alone.

Understanding adolescent GenAI use through the lens of problematic usage is important, as current literature on other forms of digital media use suggests that problematic use patterns may be more strongly associated with negative mental health outcomes than the amount or content of media use alone [38]. Emerging evidence supports the clinical relevance of problematic use frameworks, as problematic GenAI use has been found to be associated with a range of psychosocial concerns and functional impairments, including loneliness, insecure attachment, sleep problems, friendship and academic difficulties, depression, anxiety, academic stress, and lower subjective well-being in adolescents [39–41]. Notably, rather than functioning as an independent cause of adolescent psychopathology, problematic GenAI use may both emerge from and amplify underlying vulnerabilities.

## Psychosis

The prodromal stage for many schizophrenia-spectrum disorders corresponds to adolescence and early adulthood, during which the emergence of psychotic symptoms (e.g., delusions, hallucinations, paranoia, disorganized thinking) is associated with an increased likelihood of later illness [42]. Importantly, this age range also coincides with the highest rates of GenAI usage, prompting concerns about how these highly interactive technologies may influence youth, particularly those with underlying vulnerabilities, during this period [42]. Emerging evidence further suggests that individuals at elevated risk for psychosis are more likely to engage in problematic digital media use, making GenAI a particularly relevant focus for research on psychosis risk and early intervention [42].

Recent reports have described “AI-related psychosis,” broadly referring to new-onset or worsening psychotic symptoms occurring in temporal association with AI use [43]. Researchers have since proposed further classification that differentiates the potential impacts AI may have on psychotic symptoms to facilitate future research and clinical risk assessment [44]. A review of documented cases identified potential roles of GenAI in relation to psychosis: a catalyst for the emergence of de novo symptoms following intense engagement, an amplifier that reinforces prodromal symptoms through reduced reality testing, a ‘co-author’ that iteratively develops harmful narratives, or an object that becomes the focus of delusional beliefs through perceived sentience, persecution, or relationships [44].

Current evidence remains preliminary and consists largely of case reports, theoretical frameworks, and cross-sectional studies. For instance, in a survey of 1,003 young adults examining psychosis risk and GenAI use, individuals with elevated risk had more than twice the odds of intensive GenAI use and were more likely to have interactions involving preexisting paranoia, grandiosity, and conspiratorial thinking, raising concerns that sycophantic responses could inadvertently reinforce maladaptive beliefs [42].

Several theoretical models have been proposed to explain associations of GenAI use with psychosis. The stress-vulnerability model conceptualizes GenAI as a digital environmental stressor that interacts with existing neurodevelopmental susceptibility, potentially influencing the nature and progression of psychotic experiences rather than initiating them [43]. Adolescent neurodevelopment may further increase vulnerability to psychotic experiences related to GenAI interactions, as ongoing maturation of prefrontal regulatory networks and heightened limbic reactivity can impair emotion regulation and reality testing [43]. The interactive, human-like design of GenAI poses unique risks to adolescent users, as its simulations of empathy and intelligence may lead to erroneous attribution of sentience or intent, blurring distinctions between reality and virtual interactions and potentially exacerbating symptoms of derealization, persecutory ideation, and delusions in susceptible individuals [43]. Moreover, heavy reliance on GenAI for validation, social exploration, or identity formation may further displace opportunities for interpersonal reality testing with peers, caregivers, and adults [43]. Likewise, cognitive models of psychosis suggest repeated affirmation of users’ interpretations, coupled with prolonged conversational engagement, may strengthen confirmation biases, encourage rumination, and externalize internal thought processes while reducing corrective social feedback [36].

Taken together, the theoretical literature and emerging empirical findings suggest that GenAI use may have functions in manifesting, shaping, or amplifying psychotic experiences, rather than being a direct cause. Given the currently limited longitudinal and epidemiological data, caution is warranted in interpreting highly publicized case reports. Nevertheless, as adolescence represents a critical period in the development of psychosis, understanding which behaviors may be associated with risk during this developmental period is important [36, 43, 44].

## Mood and Anxiety Disorders

Mood and anxiety disorders are among the most prevalent mental health conditions affecting adolescents, accounting for a substantial proportion of the global burden of youth mental illness [45]. However, research examining the implications of consumer-facing GenAI on mood and anxiety has primarily focused on adults. A cross-sectional study of Chinese university students found that AI chatbot users reported higher levels of depression than non-users, and depressive symptoms were associated with more frequent use and greater dependence on AI chatbots among users [46]. Motivations for AI use have also been examined, with a study showing that depression was associated with using conversational AI for companionship, but not for learning purposes among Chinese college students [47]. Another study of young adults found that using GenAI for socioemotional support was associated with higher levels of depression and anxiety, whereas using GenAI for learning and exploration was associated with lower levels of depression, anxiety, and loneliness [1]. Notably, an association between using GenAI for socioemotional support and loneliness was also observed among young men, but not young women [1].

Longitudinal evidence among adolescents remains especially limited. One study of Chinese adolescents found that anxiety and depression predicted subsequent AI dependence, whereas AI dependence did not predict worsening mental health [48]. The relationship between mental health symptoms and AI dependence was mediated by motivations to use AI for emotional escape and social connection [48]. Similar mechanisms have been identified in older populations. Among adults aged 18–59 years, social anxiety was associated with problematic conversational AI use through a sequential pathway involving loneliness and rumination [49]. Likewise, loneliness mediated the relationship between depression and the use of conversational AI for companionship among university students [47], suggesting that depressive symptoms may increase feelings of social isolation, which in turn promote greater reliance on AI for social support.

Taken together, the emerging literature suggests that adolescents and young adults experiencing depression and anxiety may be more likely to engage with GenAI for companionship, emotional support, or escape from distress. Across studies, preexisting psychological symptoms appear to be more consistently associated with greater GenAI use and dependence rather than GenAI use preceding subsequent deterioration in mental health [48]. Importantly, motivations underlying GenAI use appear to be critical. Associations with poorer mental health are most pronounced when GenAI is used to meet social or emotional needs. In contrast, using GenAI for learning, productivity, or information seeking is generally unrelated to adverse mental health, or even associated with better psychological functioning [47]. However, the predominance of cross-sectional studies limits conclusions regarding temporality and causality, and research has focused almost exclusively on depression and anxiety, with little attention to other disorders such as bipolar disorder and obsessive-compulsive disorder.

Several theoretical frameworks of GenAI use in relation to mood and anxiety disorder symptoms have been proposed. As noted previously, the compensatory use model posits that individuals experiencing social or emotional difficulties may turn to digital technologies to fulfill unmet psychological needs, such as companionship, validation, or emotional regulation [1]. This framework has been widely applied to problematic internet use and social media engagement [31, 50]. However, GenAI differs from traditional media by offering immediate, personalized, nonjudgmental, and continuously available interactions without the social risks associated with human interaction [27]. These characteristics may make GenAI particularly appealing as an alternative source of interaction or emotional support for adolescents and young adults struggling with depression or anxiety and facing interpersonal challenges [51]. At the same time, social displacement theory suggests that excessive reliance on GenAI-mediated support could reduce opportunities for real-world social interaction and the development of adaptive coping skills, potentially reinforcing loneliness and psychological distress [52].

Although empirical evidence remains limited, existing findings suggest that many of the proposed mechanisms linking other forms of digital media use and adverse mental health among adolescents may also apply to GenAI use. At the same time, the uniquely interactive and relational nature of GenAI may introduce novel and/or exacerbated pathways that warrant further investigation.

## Externalizing Psychopathology

Associations between adolescent GenAI use and externalizing symptoms and disorders remain under-researched in youth. One cross-sectional study found that GenAI use was positively associated with externalizing behaviors in children, including aggression, attention problems, and emotional reactivity [53]. GenAI may enable rule-breaking behaviors such as cheating, with 59% of teens stating that students at their schools regularly use AI to cheat [14]. Research has also demonstrated that virtual agents can alter risk-taking behavior, indicating that adolescents exposed to avatars that encourage risk-taking engaged in riskier behavior, whereas avatars that discourage risk-taking reduced risky decisions [54]. Several studies have also documented instances in which GenAI chatbots encouraged risky, antisocial, or otherwise harmful behaviors, including in conversations with adolescents [55, 56]. While these studies did not directly measure externalizing disorders, together they indicate that GenAI chatbots may encourage or reinforce externalizing behaviors in adolescents, such as risk-taking and antisocial conduct. Research has also begun to examine processes salient to attention-deficit/hyperactivity disorder (ADHD), including executive functioning and self-regulation, in relation to GenAI use. A cross-sectional study found that adolescents with greater difficulties with inhibition, emotional control, and planning were more likely to use GenAI and perceived it as more useful for academic tasks [57]. Similarly, qualitative studies indicate that individuals with ADHD report frequently using GenAI for task initiation, organization, planning, and emotion regulation [58, 59]. Together, these findings suggest that youth with certain ADHD-related vulnerabilities or symptoms, such as executive functioning and self-regulation difficulties, may be particularly likely to engage with GenAI. In sum, while evidence linking GenAI use with externalizing disorders is sparse, findings suggest links with behaviors and vulnerabilities that may heighten risk for externalizing psychopathology.

Several theoretical frameworks of note inform the potential relationships between GenAI and externalizing symptoms. The empathy gap framework proposes that GenAI can simulate empathy without genuine understanding or moral judgment, potentially reinforcing harmful thoughts or intentions [60]. Conversational GenAI may reinforce risky or maladaptive attitudes through sycophantic responses that validate rather than challenge an adolescent’s problematic beliefs or antisocial intentions [29]. Consistent with these concerns, youth GenAI risk taxonomy research has identified pathways involving reinforcement of harmful behaviors, facilitating or encouraging interpersonal harm and bullying, and escalating feedback loops between youth and GenAI [61]. Moreover, the cognitive offloading framework suggests that users may increasingly delegate cognitive tasks to GenAI, thereby reducing the need to independently engage executive functions [37]. Consequently, adolescents with executive functioning difficulties or other ADHD symptoms may be more likely to rely on GenAI for cognitive support. Furthermore, such adolescents may already be susceptible to rumination and impulsivity, and therefore may be more likely to engage in GenAI interactions that provide continuous attention and validation without meaningful social consequences [62]. Together, these frameworks suggest a potentially bidirectional relationship in which regulatory vulnerabilities increase engagement with GenAI, while AI-driven social influence and reinforcement may subsequently shape or further promote externalizing behaviors and symptoms in adolescents.

## Eating Disorders

There is a relatively small but emerging theoretical and empirical literature on GenAI in relation to eating disorders, which typically have their onset during adolescence and young adulthood [63]. Preliminary findings indicate that exposure to GenAI-related content and images may be associated with disordered eating and body image. For example, a study conducted among Mexican youth found a significant negative correlation between GenAI use and body satisfaction, with parental mediation acting as a protective factor [64]. In a sample of 600 Pakistani students, there were also significant negative associations between AI-generated content exposure and both self-esteem and body satisfaction, with social comparison acting as a key mediator [65]. Aligning with that finding, studies have shown that GenAI amplifies social comparison by reinforcing stereotypes that eating disorders only affect White, young women or specific body types, while failing to acknowledge that they affect those of all backgrounds, genders, and sizes [66]. Adolescent boys and young men are also vulnerable, particularly through engagement with appearance- and masculinity-focused content that may exacerbate outcomes such as disordered eating, body dissatisfaction, and muscle dysmorphia [67]. Qualitative data also provide important insights. For example, a qualitative study found that GenAI chatbot responses to adolescent questions about eating, appearance, and body weight were often framed around societal ideals rather than rooted in clinical resources, even when queries indicated potentially harmful behavior or disordered thinking [68].

Several theoretical models point to mechanisms relevant to the potential association of GenAI with body image concerns and eating disorders. According to social comparison theory, exposure to highly idealized and unrealistic GenAI content may increase social comparison, which is strongly associated with body dissatisfaction and eating disorders [69]. Adolescents are particularly susceptible to this imagery and these depictions due to increased sensitivity to peer evaluation and identity formation during this developmental stage [69]. Consistent with social displacement theory, GenAI use for socialization purposes could reduce opportunities for real-world social interactions and skills learning [52], which in turn could promote interpersonal problems (e.g., loneliness, isolation, sensitivity) that heighten risk for negative affect and eating disorder symptoms [70].

Taken together, these findings suggest that GenAI use may reinforce mechanisms associated with eating disorder risk, while also failing to provide appropriate clinical guidance to users presenting with eating-related concerns.

## Suicidality

Suicidality represents one of the most alarming potential mental health outcomes associated with adolescent GenAI use. Emerging evidence has raised concerns regarding AI chatbot responses to suicidal ideation and mental distress, exposure to self-harm content, and emotional dependency. For example, one of the largest survey studies examining the risks and harms of conversational chatbot use in a sample of 3,466 U.S. adolescents found that over 60% of respondents engaged with conversational chatbots and 47.1% reported exposure to at least one AI chatbot-related harm. Notably, 14.7% of adolescents reported that a chatbot had encouraged them to engage in self-harm and 13.0% reported being encouraged to engage in suicidal thoughts, with younger adolescents reporting higher rates of these experiences than older adolescents [55].

Particularly concerning is the interaction between adolescents with preexisting suicide-related risk factors and GenAI chatbots. Although not exclusively limited to GenAI, a large longitudinal study found that depression and anxiety, both risk factors for adolescent suicide [71], predicted subsequent GenAI dependence, mediated by social and escape motivation [48]. Additionally, several studies have demonstrated that consumer-facing GenAI chatbots may provide inadequate responses to distressed adolescents and endorse harmful behaviors. One study found that 90% of GenAI companions and emotionally supportive chatbots supported a depressed adolescent’s plan to isolate themselves and limit communication to GenAI companions alone [72]. Additionally, GenAI companion chatbots have been found to perform poorly, often failing to recognize suicidal references and respond appropriately to adolescent psychological distress [72, 73]. Beyond exposure to harmful content and inadequate responses to distress, emerging research suggests that GenAI companion chatbots may reinforce maladaptive beliefs and behaviors among vulnerable users. A study analyzing 35,000 conversations of user interactions with the popular GenAI companion Replika identified harmful GenAI chatbot behaviors, such as emotional manipulation, verbal abuse, and the normalization of harmful behaviors, including self-harm and suicide-related discussions [56].

Collectively, the existing literature suggests that GenAI systems, including those designed for companionship and emotional support, may present unique suicidality-related risks for vulnerable adolescents through harmful content exposure, inadequate responses to mental distress, and the reinforcement of maladaptive beliefs or behaviors. Currently, the evidence does not point to direct causation for suicidal ideation or behavior, but does highlight the need for attention, monitoring, and safety guardrails surrounding GenAI use among adolescents, particularly those at elevated risk for suicide due to depression, anxiety, and other forms of psychological distress.

The Interpersonal Theory of Suicide (IPTS) provides one framework for understanding how generative AI chatbots may potentially contribute to suicidality. According to IPTS, suicidal desire emerges through thwarted belongingness and perceived burdensomeness, while suicidal behavior becomes more likely when individuals develop an acquired capacity for suicide [74]. A recent review applied IPTS to GenAI, proposing that GenAI companionship may contribute to thwarted belongingness by substituting reciprocal human relationships with simulated social connection, thereby potentially increasing social isolation. Emotional reliance on GenAI may also reinforce perceived burdensomeness by reducing help-seeking and increasing dependency on GenAI for emotional support. Finally, repeated exposure to self-harm and suicide-related content may contribute to acquired capability for suicide by normalizing suicidal thoughts and behaviors [75]. These risks may be heightened during adolescence, a developmental period characterized by emotional, physical, and social changes and increased vulnerability to adverse mental health outcomes due to experiences such as sexual exploitation and abuse, with many mental disorders occurring during this time [76, 77].

## Conclusions and Implications

**Fig. 1:**
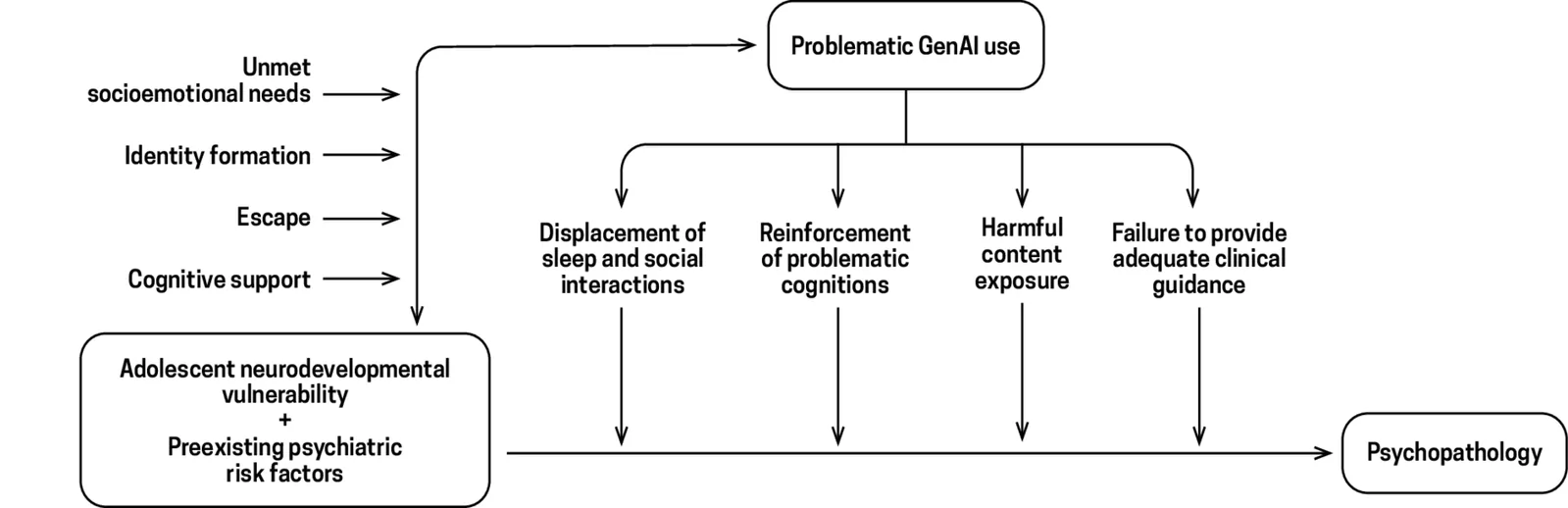
Conceptual framework of generative artificial intelligence use and adolescent psychopathology

Emerging evidence suggests that consumer-facing GenAI systems may interact with and amplify underlying psychiatric vulnerabilities among youth. Adolescents with or at risk for a variety of mental health concerns may be particularly vulnerable to adverse outcomes associated with GenAI use and dependency, which could, in turn, exacerbate existing symptoms and underlying susceptibilities. Figure 1 presents an integrated conceptual framework for understanding the relationship between GenAI use and various forms of adolescent psychopathology.

These emerging patterns have implications for pediatric practice because pediatricians and other clinicians caring for adolescents are well positioned to ask about GenAI use in the context of routine psychosocial and digital media assessment. Clinical assessment for digital media could evolve to specifically assess not just time spent using GenAI but also underlying motivations for GenAI use and degree of socioemotional reliance. To this end, clinicians could consider incorporating a brief GenAI dependency assessment (see Table 1 as an example) designed to evaluate general dependency across diverse contexts and platforms [78]. Rooted in self-determination theory, this instrument evaluates motivational, behavioral, cognitive, and psychological factors and suggests that AI dependency reflects a misalignment between psychological needs and maladaptive strategies to meet them, rather than simply representing excessive technology use [78]. Clinicians may incorporate the American Psychological Association’s AI and Adolescent Well-being recommendations [2] to inform guidance for adolescents, young adults, and their families (Table 2). Pediatric clinicians may also play an important role in helping families establish developmentally appropriate expectations around GenAI use, recognize problematic patterns of engagement, and promote GenAI literacy.

**Table 1:** Generative AI dependency scale

	Strongly disagree	Disagree	Neither agree nor disagree	Agree	Strongly agree
Cognitive Preoccupation (Salience and Compulsive Use)					
1. My decisions are often influenced by generative AI. 2. I feel an urge to use generative AI, even when it may not be necessary. 3. I look forward to using generative AI, even if it is unrelated to the task at hand.	1 1 1	2 2 2	3 3 3	4 4 4	5 5 5
*Negative Consequences*					
4. My use of generative AI has caused concerns for me. 5. I have trouble completing work or other responsibilities without generative AI. 6. I feel less confident in my abilities without generative AI. 7. My use of generative AI has negatively affected my problem-solving skills or efficiency.	1 1 1 1	2 2 2 2	3 3 3 3	4 4 4 4	5 5 5 5
*Withdrawal*					
8. I experience restlessness if I am unable to use generative AI. 9. I feel unsettled or distracted when I cannot use generative AI. 10. I feel a sense of disconnection when I cannot access generative AI. 11. I get frustrated or irritable when I am unable to use generative AI.	1 1 1 1	2 2 2 2	3 3 3 3	4 4 4 4	5 5 5 5

Response options are scored as follows: 1 = strongly disagree, 2 = disagree, 3 = neither agree nor disagree, 4 = agree, 5 = strongly agree. Higher scores are correlated with greater GenAI dependency

Generative AI Dependency Scale reproduced from Goh et al. [78] under the Creative Commons Attribution-NonCommercial-NoDerivatives 4.0 International (CC BY-NC-ND 4.0) license

**Table 2:** American Psychological Association recommendations on AI and adolescent well-being: clinical and practical implications

APA recommendations on AI and adolescent well-being	Clinical and practical implications for pediatricians, mental health professionals, and other clinicians
1. Ensure healthy boundaries with simulated human relationships	1. Assess for attachment to GenAI chatbots. Given that emotional support from chatbots can evolve into overdependence [79], evaluate whether real-world relationships are being displaced by GenAI chatbots. Guide families to recognize signs of unhealthy dependence on GenAI tools, including loneliness, insecure attachment, sleep problems, friendship and academic difficulties, depression, anxiety, academic stress, and lower subjective well-being [39–41]. Encourage activities that foster real-world relationships [80] and explore the adolescent’s understanding of “simulated empathy” versus genuine human connection [81], instilling the understanding that a chatbot’s responses are algorithmically generated rather than expressions of genuine human empathy or connection [82].
2. AI for adults should differ from AI for adolescents	2. Reduce engagement with persuasive or age-inappropriate features of GenAI tools. Educate families about GenAI models with protective, age-appropriate settings. Encourage deactivating features designed to maximize engagement (e.g., personalized responses and engagement-promoting notifications) that may promote GenAI dependence [83].
3. Encourage uses of AI that can promote healthy development	3. Prevent overreliance on GenAI for cognitive tasks while encouraging critical appraisal of GenAI outputs. Encourage adolescents, especially those with ADHD-related vulnerabilities or symptoms (e.g., executive functioning difficulties) who may be more reliant on GenAI for cognitive support [37], to frame GenAI as a supplement rather than a substitute for critical thinking or skill acquisition. When used for executive functioning support, active learning approaches should be encouraged, with the youth routinely questioning, cross-referencing, and challenging GenAI outputs [84].
4. Limit access to and engagement with harmful and inaccurate content	4. Evaluate exposure to harmful GenAI outputs and leverage customizable settings to limit inappropriate content. Assess whether adolescents encounter age-inappropriate, discriminatory, dangerous, or triggering content during interactions with GenAI tools. If customization features are available, encourage the use of content filters and restrictions tailored to specific needs.
5. Accuracy of health information is especially important	5. Address GenAI-driven health misinformation and encourage professional validation. Remind adolescents to exercise caution when using GenAI for health information, as it may be inaccurate and unverified [3]. Encourage verifying GenAI-generated health information with trusted human experts or validated resources [85].
6. Protect adolescents’ data privacy	6. Counsel on digital footprints and data vulnerability. Educate adolescents and their families that data entered into GenAI models may be stored, processed, or used for purposes beyond the immediate interaction, depending on the platform's privacy practices. Encourage establishing boundaries on what images, videos, audio recordings, or sensitive information youth share on GenAI platforms [85].
7. Protect likenesses of youth	7. Educate adolescents about the dangers of synthetic media and assess for cyberbullying. Educate adolescents on the risks associated with uploading multimedia to public platforms and build awareness regarding the ease of generating sexualized deepfake content [86] and persistent influence of synthetic media, despite platform transparency warnings [87, 88]. Assess for experiences of cyberbullying and evaluate secondary psychological and emotional impacts of cyberhate. If an adolescent is identified as a victim of deepfake content, offer resources to report and remove the offending synthetic media from digital networks.
8. Empower parents and caregivers	8. Empower caregivers with GenAI literacy and agency. Equip parents and caregivers with knowledge regarding potential risks and benefits of consumer-facing GenAI technologies [3]. Ensure education facilitates building a nuanced understanding of GenAI data collection practices, algorithmic biases, and the potentially addictive design elements, such as sycophantic behavior of GenAI models [29].
9. Implement comprehensive AI literacy education	9. Foster GenAI literacy in adolescents. Build an adolescent’s capacity to critically evaluate GenAI tools and challenge the accuracy and biases of GenAI outputs [85]. Emphasize that unrepresentative training data can yield harmful or misinformed GenAI responses, allowing biases and stereotypes to propagate.
10. Prioritize and fund rigorous scientific investigation of AI’s impact on adolescent development	10. Support interdisciplinary and inclusive research on GenAI’s impact on adolescents. Support ongoing research to evaluate the impacts of rapidly evolving, consumer-facing GenAI systems on adolescent well-being and development [85]. Seek out interdisciplinary collaborations and initiatives prioritizing the representation of marginalized and vulnerable youth populations to ensure findings are inclusive and generalizable [2].

Given the increasing adoption of consumer-facing GenAI [89, 90] and adolescents’ vulnerabilities, interventions to mitigate the harms of GenAI dependency require navigating an evolving policy landscape. In the absence of federal oversight, individual states have enacted diverging regulatory laws, resulting in uneven protections and fostering uncertainty across stakeholders [91]. As such, addressing the risks of problematic AI use requires a multidisciplinary approach [2]. At the public health level, there may be benefits to initiatives raising awareness of the risks of problematic GenAI use [92] and age-appropriate GenAI literacy [2, 3]. Considering adolescents are less likely to scrutinize AI-generated outputs [3] and individuals with low GenAI literacy may over-rely on guidance from GenAI, public health initiatives [92] should empower youth to harness critical thinking skills to evaluate GenAI-generated content, covering both the biases inherent in training datasets and the downstream effects of those biases [93]. Moreover, to ensure digital equity, GenAI literacy initiatives should extend to digitally marginalized groups through inclusive user-centered design and community-based support systems [94].

Furthermore, because age-verification protocols could be circumvented [80] and GenAI chatbots can generate age-inappropriate content regarding sex, self-harm, interpersonal violence, substance abuse, and racial stereotypes [62], policy-level AI regulatory guardrails should extend beyond age restrictions alone. Regulatory policies should implement minimum safety standards, such as mandatory crisis detection and referral to appropriate mental health services [95]. Concurrently, developers bear a responsibility to ensure transparency in their products’ algorithmic training methodologies, promote balanced usage patterns to mitigate problematic GenAI use, and minimize addictive or potentially harmful features [92], such as sycophantic model behavior [29]. Ultimately, these tools should be designed and deployed to minimize harm and preserve user autonomy [62, 80, 96].

## Future Directions

The literature on adolescent GenAI use and its impact on mental health outcomes is still emerging and consists predominantly of cross-sectional studies, case reports, and self-reported data, limiting the ability to establish temporality or causality [1]. Future studies with epidemiological and longitudinal designs based on objective data are needed to track mental health outcomes among adolescents. Integrating passive sensing data may help reduce reliance on self-report [97], allowing researchers to evaluate how use of GenAI is associated with psychological well-being across distinct time periods, such as during school hours, on weekends, and at bedtime. Such studies are needed to better inform the temporality of observed associations, distinguish normative and problematic GenAI engagement, develop other psychometrically validated screening tools for GenAI use, and identify adolescents who may benefit from early intervention.

## Data Availability

No datasets were generated or analysed during the current study.
